# Radiotherapy-induced oxidative stress and fibrosis in breast cancer are suppressed by vactosertib, a novel, orally bioavailable TGF-β/ALK5 inhibitor

**DOI:** 10.1038/s41598-022-20050-9

**Published:** 2022-09-27

**Authors:** Jiyoung Park, Jiwon Choi, Ilyoung Cho, Yhun Yhong Sheen

**Affiliations:** 1 College of Pharmacy, Ewha Womans University, 52, Ewhayeodae-gil, Seodaemun-gu, Seoul, 03760 Republic of Korea; 2 Present Address: National Center for Efficacy Evaluation for Respiratory Disease Products, Korea Institute of Toxicology, 30 Baehak1-gil, Jeongeup, Jeollabuk-do 56212 Republic of Korea

**Keywords:** Cancer, Oncology

## Abstract

Radio-resistance resulting from radiotherapy-induced fibrosis is a major clinical obstacle in breast cancer treatment because it typically leads to cancer recurrence, treatment failure, and patient death. Transforming growth factor-β (TGF-β) is a key signal messenger in fibrosis, which plays an important role in radiation-induced fibrosis and cancer stem cell (CSC) development, may be mediated through the generation of oxidative stress. This study was conducted to confirm the efficacy of vactosertib, a TGF-β/ALK5 inhibitor, as a potent inhibitor in radiation-induced oxidative stress generation, fibrosis and CSC development. We used a 4T1-Luc allograft BALB/c syngeneic mouse model and 4T1-Luc and MDA-MB-231 cells for histological analysis, qRT-PCR, western blotting, ROS analysis, mammosphere formation analysis, monolayer fluorescence imaging analysis. Radiotherapy induces TGF-β signaling, oxidative stress markers (4-HNE, NOX2, NOX4, PRDX1, NRF2, HO-1, NQO-1), fibrosis markers (PAI-1, α-SMA, FIBRONECTIN, COL1A1), and CSC properties. However, combination therapy with vactosertib not only inhibits these radiation-induced markers and properties by blocking TGF-β signaling, but also enhances the anticancer effect of radiation by reducing the volume of breast cancer. Therefore, these data suggest that vactosertib can effectively reduce radiation fibrosis and resistance in breast cancer treatment by inhibiting radiation-induced TGF-β signaling and oxidative stress, fibrosis, and CSC.

## Introduction

Radiotherapy is a major treatment modality for a wide range of cancers. Today, over 60% of all cancer patients undergo radiotherapy, either alone or, more commonly, in combination with surgery and/or chemotherapy^1,2^. However, breast cancer radiotherapy inevitably involves some limitations like tissue injury and radiation-induced fibrosis that leads to radio-resistance and cancer recurrence, possibly resulting in treatment failure and patient death^3^. Normal tissue damage and fibrosis are caused by increasing radiation by cancer cell resistance that limits the delivery of sufficient doses of radiation^4^. Fibrosis is an unusual tissue stiffness caused by abnormal accumulation of collagen in the extracellular matrix (ECM) that results in tissue destruction and organ dysfunction^4,5^. Since radiation-induced fibrosis significantly impacts quality of patient life, and acquired radio-resistance is a major clinical obstacle for breast cancer patients receiving radiotherapy, it is important to find a therapeutic strategy to mitigate the risk of radio-resistance^3,6^.

Evidence shows that radiation-induced transforming growth factor-β (TGF-β) stimulates major events in tissue fibrosis, such as reactive oxygen species (ROS) generation, myofibroblast and fibrocyte activation, and synthesis of ECM components^7,8^. TGF-β plays a pivotal role in fibrosis through various signaling pathways. The canonical (Smad-dependent) TGF-β pathway including TGF-β type I receptor (activin receptor-like kinase 5, ALK5) phosphorylation of Smad2/3 (p-Smad2/3) is one of the most common signaling pathways that mediate progression of radiation-induced fibrosis by increasing the expression of *Collagen I* and *III*, *Vimentin*, and *α-SMA* after exposure to radiation^4,5^. TGF-β promotes ROS generation by diverse mechanisms, and ROS stimulates the production of *Collagen I*, up-regulates TGF-β, and supports radiation-induced fibrosis^4,8^. In light of these findings, TGF-β signaling is a potential target for the prevention of radiotherapy-induced fibrosis and radio-resistance. Therefore, highly specific, selective TGF-β/ALK5 inhibitors that block the binding of TGF-β to its receptor are researched to prohibit TGF-β signaling.

Among these, vactosertib (EW-7197) is an orally bioavailable TGF-β/ALK5 inhibitor that selectively blocks TGF-β signaling and targets ALK5 with high potency^9,10^. Vactosertib is rapidly absorbed after oral dosing and has shown high efficacy with low toxicity in several animal models^8,11–13^. Vactosertib shows potential as an anti-fibrosis agent by inhibition of Smad-dependent TGF-β signaling and decrease of oxidative stress in hepatic, renal, and pulmonary fibrosis rat and mouse models^8^. Vactosertib also shows antifibrotic properties by weakening TGF-β/Smad signaling and reducing oxidative stress in cholestatic liver fibrosis rat and ulcerative colitis mouse models^14,15^. Furthermore, vactosertib suppresses paclitaxel-induced fibrotic markers by weakening paclitaxel-induced ROS and inhibits lung metastasis in a paclitaxel-treated mouse model of breast cancer^13^. Therefore, vactosertib combination with chemotherapy or radiotherapy could be a candidate for breast cancer therapy considering its critical role in anti-fibrosis. Based on the promising pharmacokinetic, pharmacologic, and toxicologic aspects of vactosertib, a first-in-human phase I study was conducted to characterize the population pharmacokinetics in patients with advanced stage solid tumors^10,16^. Today, clinical studies of vactosertib in combination with several chemotherapies are being conducted in patients with various cancer types, such as gastric cancer (NCT03698825), non-small cell lung cancer (NCT03732274), colorectal or gastric cancer (NCT03724851), desmoid tumor (NCT03802084), and multiple myeloma (NCT03143985).

In this study, we hypothesized that vactosertib would repress fibrosis and oxidative stress generation by inhibiting TGF-β/Smad signaling. We begin the examination of whether vactosertib shows favorable therapeutic effects in breast cancer patients undergoing radiotherapy by investigating the effects in an irradiated mouse model of breast cancer. We found that blocking radiation-induced TGF-β signaling with an ALK5 inhibitor, vactosertib, can suppress radiation-induced oxidative stress generation, fibrosis, and cancer stem cell (CSC) development in breast cancer treatment.

## Results

### Vactosertib, inhibiting TGF-β/Smad signaling, regulates the fibrosis marker

**Figure 1:**
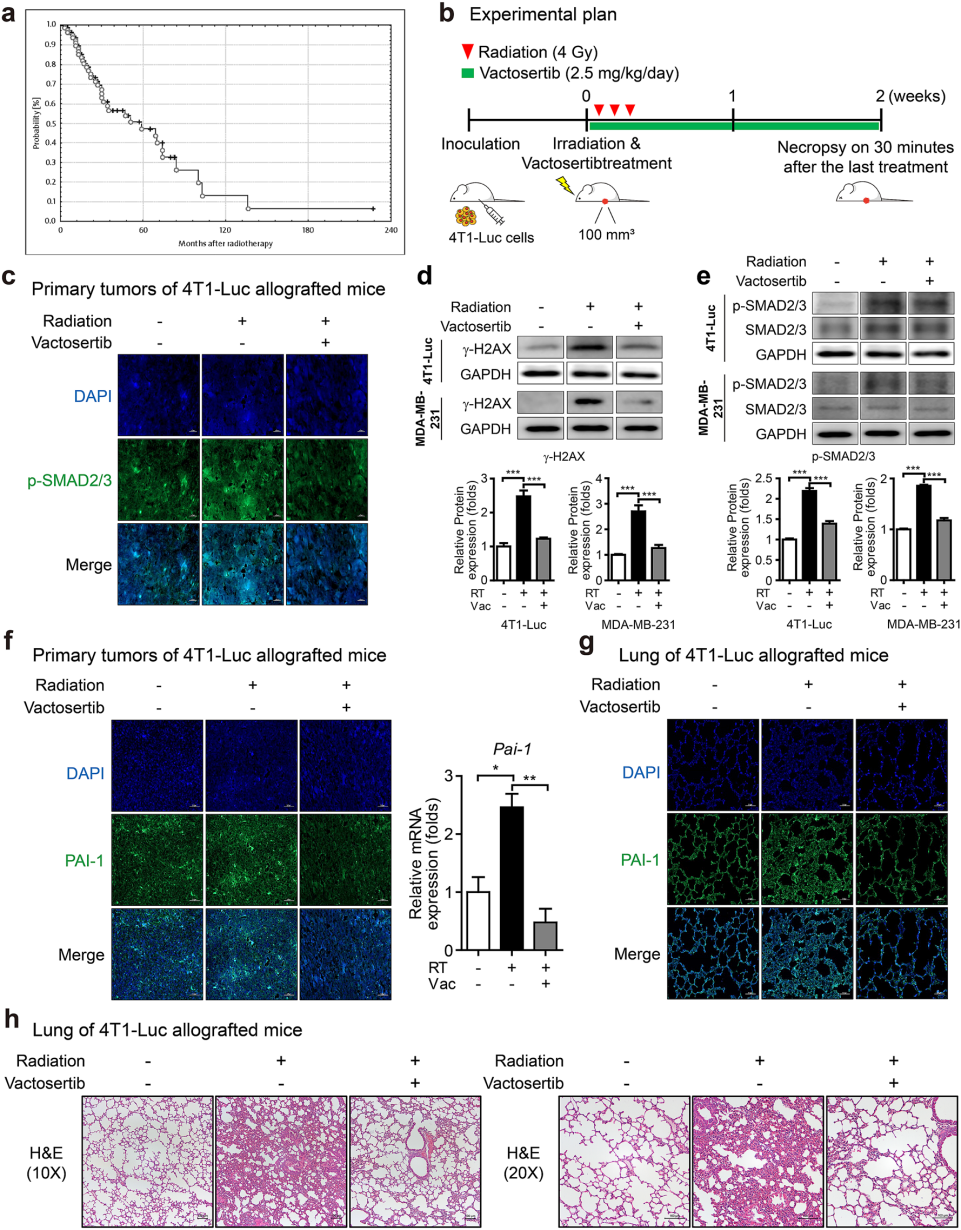
Effect of vactosertib as a treatment strategy for radiation-induced fibrosis. (**a**) A Kaplan–Meier plot of the probability of being unaffected by fibrosis (> grade III) after radiotherapy. The risk of fibrosis estimated in 86 breast cancer patients increased with time after radiotherapy. Reprinted by permission from Johannes Claßen MD et al.: Springer Nature, Strahlentherapie und Onkologie^17^, (Fibrotic Changes after Postmastectomy Radiotherapy and Reconstructive Surgery in Breast Cancer, Johannes Claßen MD et al.), Copyright, (2010). (**b**) Scheme of the experimental mouse model of breast cancer for co-treatment with vactosertib and radiation. Mice were injected with 4T1-Luc cells and were divided randomly into three groups when tumor volume was 70–100 mm^3^. Mice were treated with vactosertib 2.5 mg/kg *p.o*. for two weeks and were concurrently irradiated with 4 Gy/day over their whole body for three days. (**c**) Protein expression of p-SMAD2/3 by fluorescence immunohistochemistry analysis in irradiated primary tumors of mice (× 40, scale bar: 20 μm). In confocal images, p-SMAD2/3 emitted bright green fluorescence. (**d, e**) Protein expression of γ-H2AX (**d**) and p-SMAD2/3 (﻿**e**) in 4T1-Luc and MDA-MB-231 cells by western blot analysis (Band was normalized by GAPDH.). The band image was cropped after selecting a representative blot, and the sample was derived from the same experiment. Original blots are presented in Supplementary Fig. 1. (**f**) Fluorescence immunohistochemistry analysis and quantitative reverse transcription-polymerase chain reaction (qRT-PCR) analysis of PAI-1 in irradiated primary tumors of mice (Relative mRNA expression was normalized by *Ppia*.). Expression of PAI-1 was shown by green fluorescence image (× 20, scale bar: 50 μm). (**g**) Fluorescence immunohistochemistry analysis of PAI-1 in the lung of mice. Expression of PAI-1 was shown by green fluorescence image (× 20, scale bar: 50 μm). (**h**) H&E staining in the lungs of mice is displayed as images showing the morphology of the lungs and the degree of fibrosis by phase-contrast microscopy (× 10 and 20, scale bar: 100 μm).

We reviewed clinical data that evaluated the risk of fibrosis in breast cancer patients after radiotherapy to better understand the need for a new strategy to relieve radiation-induced fibrosis. In a retrospective study of 86 breast cancer patients treated with breast reconstruction and radiotherapy, the incidence of fibrosis (> grade III) of the reconstructed breast at three years after radiotherapy was 43% (95% confidence interval 30–65%)^17^. Furthermore, the Kaplan–Meier plot for the probability of fibrosis (> grade III) after radiotherapy showed that the risk increased with time (Fig. 1a) ^17^. Another retrospective study consistently reported that the incidence rate of grade III fibrosis of the reconstructed breast at 38 months after radiotherapy was 33.3% in 69 breast cancer patients with breast reconstruction^18^. These studies revealed that radiation-induced fibrosis would impact the long-term outcomes of breast reconstruction and lower the quality of life in patients. Therefore, a new therapeutic strategy to prevent radiation-induced fibrosis is needed.

We established a 4T1-Luc allograft BALB/c syngeneic mouse model to investigate whether vactosertib blocks radiation-induced TGF-β signaling in breast cancer (Fig. 1b). A radiation dose of 4 Gy × 3 reduced tumor volume in mice, and a dose of 2.5 mg/kg of vactosertib did not show organ toxicity to mice^19^. The protein level of p-SMAD2/3 was increased in the irradiated primary tumor but was decreased by combination treatment with vactosertib and radiation (Fig. 1c). DNA damage caused by irradiation was confirmed through the expression of γ-H2AX, and it was suppressed by co-treatment with vactosertib (Fig. 1d). The protein level of p-SMAD2/3 was increased in irradiated breast cancer cell lines but was decreased by combination treatment with vactosertib and radiation (Fig. 1e). In accordance with in vivo data, radiation-induced the protein level of p-SMAD2/3, and co-treatment with vactosertib compensated for this effect in 4T1-Luc and MDA-MB-231 cells according to western blot assay.

To investigate the inhibitory effect of vactosertib on radiation-induced fibrosis, we identified the regulation of fibrosis markers both in vivo and in vitro. Regulation of the expression of *Pai-1*, a profibrotic gene induced by TGF-β, is mediated by Smad3 activation through ALK5^20^. Fluorescence IHC and quantitative reverse transcription-polymerase chain reaction (qRT-PCR) were carried out to examine whether vactosertib blocks induction of PAI-1. We found an increased protein level of PAI-1 in irradiated primary tumors of 4T1-Luc allografted mice but a decreased level in mice treated with vactosertib combined with radiation. Along with this result of fluorescence IHC, the mRNA expression of *Pai-1* by qRT-PCR showed an increase that was significantly abolished by co-treatment with vactosertib (Fig. 1f). Moreover, vactosertib treatment decreased the elevated protein level of PAI-1 in the lung tissue of irradiated 4T1-Luc allografted mice (Fig. 1g). However, the degree of decrease of PAI-1 protein in the lung tissue was less than that in the primary tumor. To further investigate radiation-induced lung fibrosis, H&E staining was performed on mouse lung tissue. Lung tissue fibrosis was induced by radiation, and combination treatment with vactosertib attenuated radiation-induced fibrosis (Fig. 1h).

**Figure 2:**
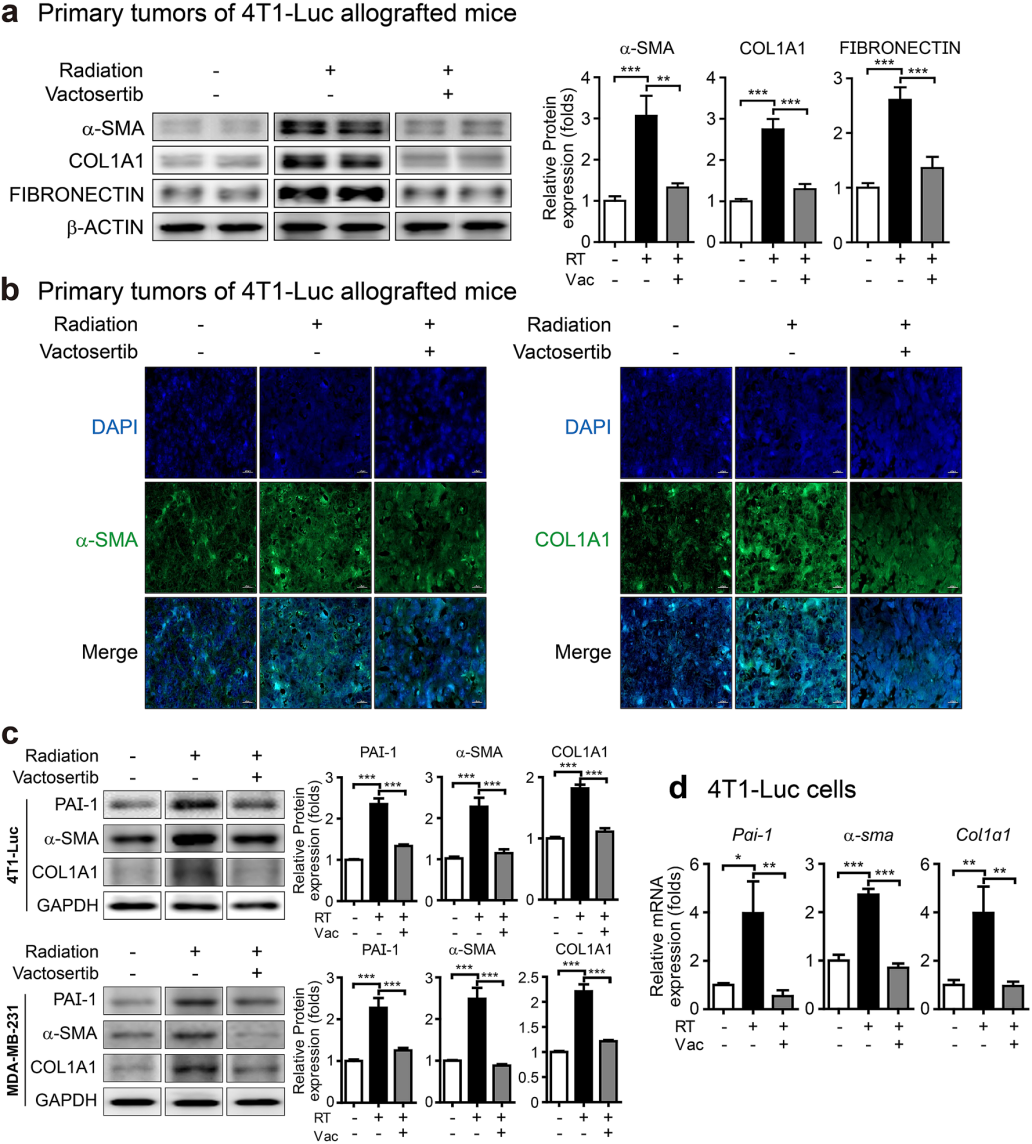
Vactosertib regulates the radiation-induced fibrosis markers. (**a**) Protein expression of α-SMA, COL1A1, FIBRONECTIN in irradiated primary tumors of mice with or without co-treatment with vactosertib (Band was normalized by β-ACTIN.). Original blots are presented in Supplementary Fig. 2. (**b**) Images by fluorescence immunohistochemistry analysis of α-SMA and COL1A1 in irradiated primary tumors of mice. Expression of α-SMA and COL1A1 was shown by green fluorescence image (× 40, scale bar: 20 μm). (**c**) Protein expression of PAI-1, α-SMA, and COL1A1 in 4T1-Luc and MDA-MB-231 cells by western blot analysis (Band was normalized by GAPDH.). The band image was cropped after selecting a representative blot, and the sample was derived from the same experiment. Original blots are presented in Supplementary Fig. 1. (**d**) mRNA expression of *Pai-1*, *α-sma*, and *Col1a1* in 4T1-Luc cells (Relative mRNA expression was normalized by *Ppia*.).

Differentiation of fibroblastic cells into myofibroblasts, which are the major source of ECM components and are considered significant effector cells of tissue fibrosis, is a primary process in the development of tissue fibrosis, and TGF-β induces this process^21^. α-Smooth muscle actin (α-SMA, a marker for a subset of myofibroblasts), FIBRONECTIN (an ECM component), and COL1A1 (a gene that produces a component of type I collagen) are used as markers for fibrosis^21,22^. Western blot analysis confirmed that the protein expression of α-SMA, COL1A1, and FIBRONECTIN was induced in irradiated primary tumors of 4T1-Luc allografted mice, and these increased markers were significantly decreased by co-treatment with vactosertib (Fig. 2a). These results were confirmed by fluorescence IHC analysis showing that the elevated protein levels of α-SMA and COL1A1 were reduced by vactosertib in irradiated primary tumors of 4T1-Luc allografted mice (Fig. 2b). We also conducted in vitro assays in several breast cancer cell lines to clarify the impact of radiation and vactosertib on fibrosis markers. Radiation-induced increases in protein levels of PAI-1, α-SMA, and COL1A1 in 4T1-Luc and MDA-MB-231 cells were considerably reduced by co-treatment with vactosertib (Fig. 2c). Furthermore, the mRNA expression of these markers was increased in irradiated 4T1-Luc cells and was significantly decreased by co-treatment with vactosertib (Fig. 2d). Based on these findings, the TGF-β pathway is a potential target for prevention of radiation-induced fibrosis, and vactosertib appears to be a promising ALK5 inhibitor.

### Vactosertib suppresses oxidative stress generation

**Figure 3:**
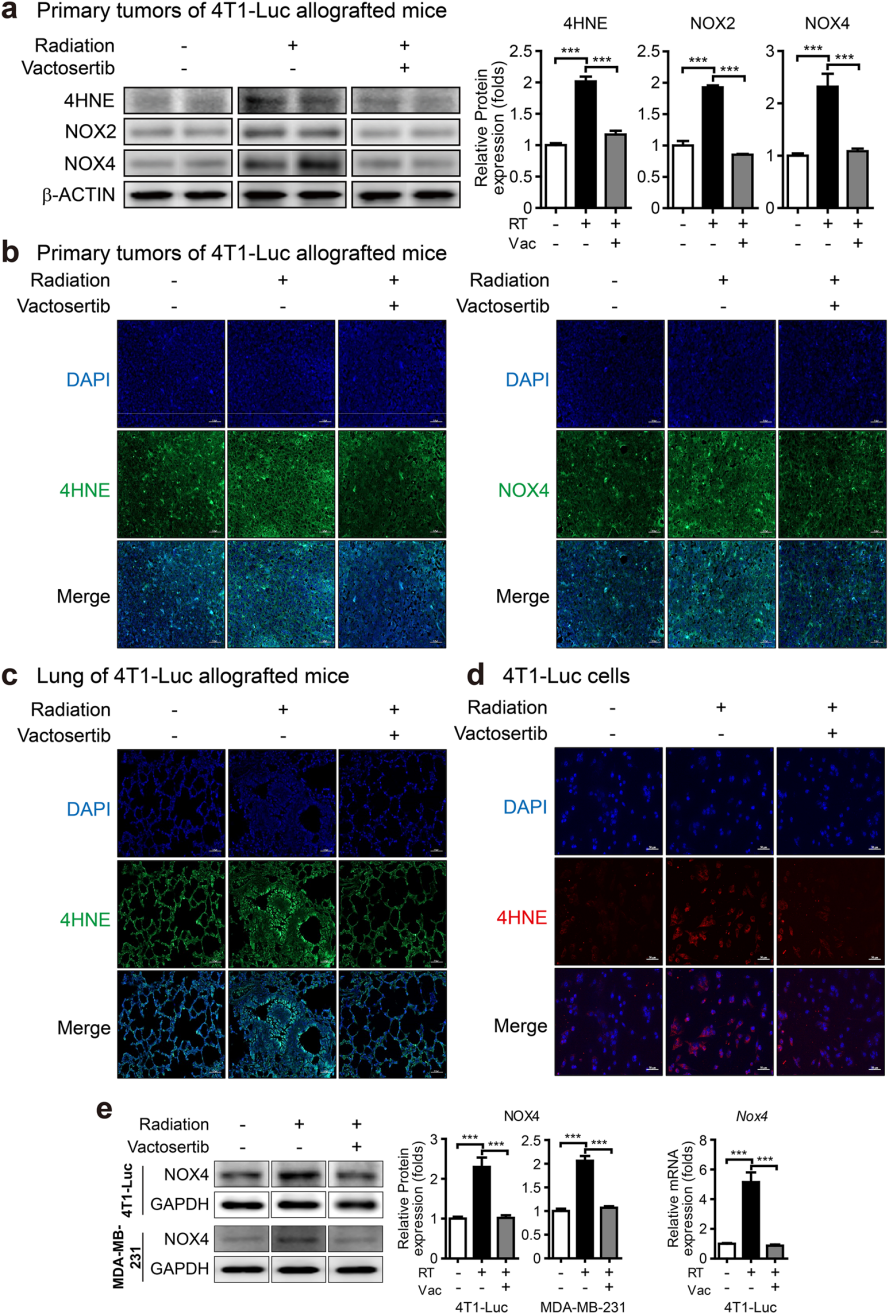
Vactosertib suppresses lipid peroxidation and NADPH oxidases (NOXes). (**a**) Protein expression of 4-HNE, NOX2, NOX4 in irradiated primary tumors of mice with or without co-treatment with vactosertib (Band was normalized by β-ACTIN.). Original blots are presented in Supplementary Fig. 2. (**b, c**) Fluorescence immunohistochemistry analysis of 4-HNE and NOX4 in irradiated primary tumors (**b**) and 4-HNE in the lung (**c**). Expression of 4-HNE and NOX4 was shown by green fluorescence image (× 20, scale bar: 50 μm). (**d**) Immunofluorescence staining of 4-HNE in irradiated 4T1-Luc cells. Expression of 4-HNE was shown by red fluorescence image (× 20, scale bar: 50 μm). (**e**) Protein expression of NOX4 in 4T1-Luc and MDA-MB-231 cells by western blot analysis (Band was normalized by GAPDH.). The right graph shows the relative intensity of mRNA expression of *Nox4* in 4T1-Luc cells. The band image was cropped after selecting a representative blot, and the sample was derived from the same experiment. Original blots are presented in Supplementary Fig. 1.

Oxidative stress plays a major role as a microenvironment factor in tissue fibrosis development^8^. 4-Hydroxy-2,3-nonenal (4-HNE), an aldehyde product of lipid peroxidation, is used as a marker of ROS and a measure of pathological changes under oxidative stress^8,13,23^. We found that elevation of 4-HNE level in vivo was decreased by treatment with vactosertib in western blot and fluorescence IHC analyses (Fig. 3a, b). Furthermore, vactosertib decreased the elevated level of 4-HNE in the lung tissue of irradiated 4T1-Luc allografted mice (Fig. 3c). We also detected 4-HNE in 4T1-Luc cells by immunofluorescence (IF) staining and found that vactosertib prevented the increase of 4-HNE induced by radiation (Fig. 3d).

TGF-β increases the generation of ROS, and oxidative stress mediates the fibrogenic effect of TGF-β^24^. In TGF-β-induced fibrogenic disorders, NADPH oxidases (NOX family members) generate ROS, resulting in fibrosis by activation of myofibroblasts and generation of ECM proteins^25,26^. Western blot analysis confirmed that the expression of NOX2 and NOX4, the major subtypes responsible for ROS production, was increased in vivo due to radiation and was significantly decreased by vactosertib (Fig. 3a). This effect of vactosertib was confirmed for the protein level of NOX4 measured by fluorescence IHC analysis (Fig. 3b). In vitro, the expression of NOX4 was significantly induced by radiation and was considerably reduced by vactosertib treatment in both irradiated 4T1-Luc and MDA-MB-231 cells by western blot analysis. It also was confirmed by qRT-PCR analysis in irradiated 4T1-Luc cells (Fig. 3e).

**Figure 4:**
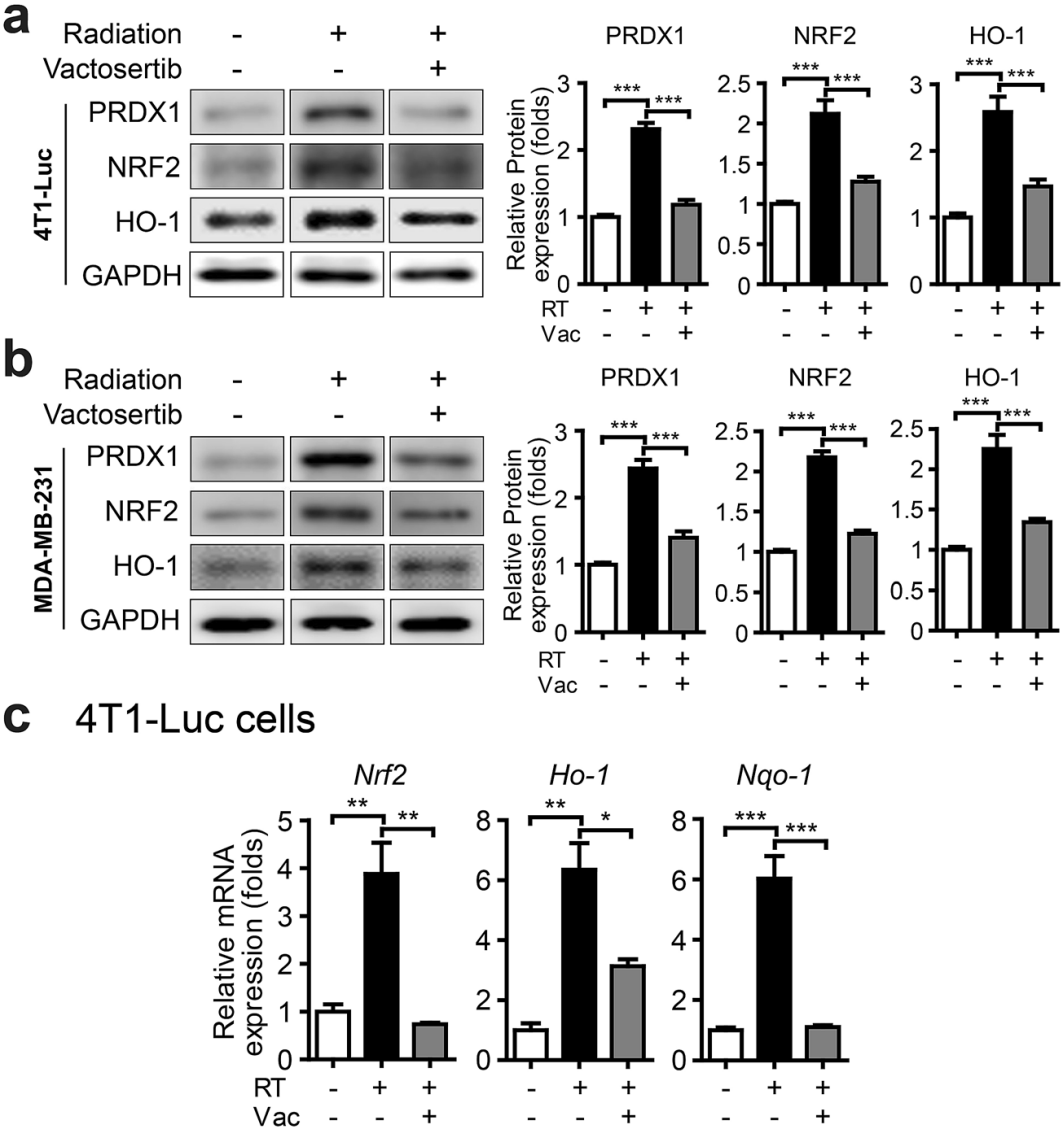
Vactosertib blocks radiation-induced ROS markers. (**a, b**) Protein expression of PRDX1, NRF2, HO-1 in 4T1-Luc cells (**a**) and MDA-MB-231 cells (**b**) by western blot analysis (Band was normalized by GAPDH.). The band image was cropped after selecting a representative blot, and the sample was derived from the same experiment. Original blots are presented in Supplementary Fig. 1. (**c**) mRNA expression of *Nrf2*, *Ho-1*, and *Nqo-1* in 4T1-Luc cells (Relative mRNA expression was normalized by *Ppia*.).

Peroxiredoxins (PRDXs), a family of peroxidases that reduce peroxides, are induced by oxidative stress and are critical protectors against oxidative damage^27–29^. Nuclear factor erythroid 2-related factor 2 (NRF2) plays a significant role in the regulation of ROS as a transcription factor by promoting antioxidant proteins, such as heme oxygenase (HO-1) and NAD(P)H:quinone oxidoreductase (NQO1)^30^. We observed that the protein levels of PRDX1, NRF2, and HO-1 were increased in irradiated 4T1-Luc and MDA-MB-231 cells, but this effect was weakened when cells were irradiated in combination with vactosertib (Fig. 4a, b). We also determined the mRNA expression levels of *Nrf2* and *Nrf2*-dependent genes (*Ho-1, Nqo-1*) to investigate whether vactosertib blocks radiation-induced ROS. In vitro, radiation up-regulated the expression of *Nrf2*, *Ho-1*, and *Nqo-1*, but vactosertib attenuated their expression in 4T1-Luc cells (Fig. 4c).

**Figure 5:**
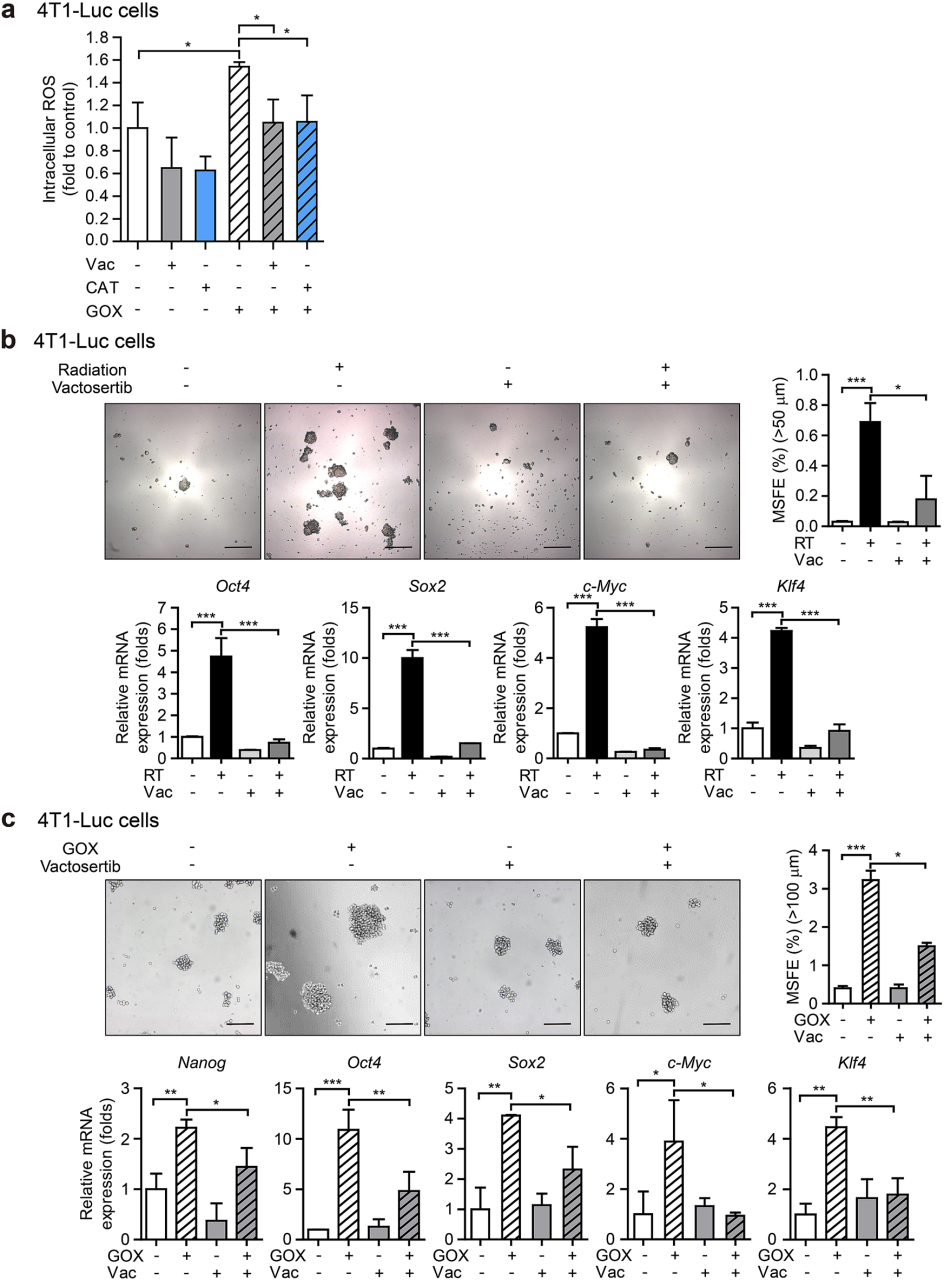
Vactosertib mitigates radiation-induced cancer stem cell properties. (**a**) Effect of vactosertib on reactive oxygen species (ROS) produced by GOX in 4T1-Luc cells. The graph shows the level of glucose oxidase (GOX)-induced DCF-sensitive ROS by Catalase (CAT) or vactosertib treatment. (**b**) Radiation-induced pluripotent stem cell regulators (*Oct4*, *Sox2*, *c-Myc*, *Klf4*) and mammosphere forming efficiency (MSFE) of 4T1-Luc cells (Relative mRNA expression was normalized by *Hprt*.). The upper panels show the phase-contrast microscopic images of spheres after seven days of culture in the ultra-low attachment condition (× 5, scale bar: 100 μm). The right graph shows the MSFE based on the number of spheres (> 50 μm). (**c**) GOX-induced pluripotent stem cell regulators (*Nanog*, *Oct4*, *Sox2*, *c-Myc*, *Klf4*) and MSFE of 4T1-Luc cells (Relative mRNA expression was normalized by *Ppia*.). The upper panels show the phase-contrast microscopic images of spheres after seven days of culture in the ultra-low attachment condition (× 5, scale bar: 100 μm). The right graph shows the MSFE based on the number of spheres (> 100 μm).

To further investigate whether vactosertib not only inhibits intracellular ROS generated through TGF-β-dependent NOX expression but also blocks ROS generated through intracellular oxidoreductase, we evaluated the effect of GOX-induced ROS inhibition in 4T1-Luc cells. Similar to the effect of the potent H_2_O_2_ degrading enzyme, Catalase (CAT), vactosertib inhibited the level of ROS increased by GOX (Fig. 5a). These results suggest that vactosertib is an effective inhibitor of ROS.

### Vactosertib mitigates radiation-induced cancer stem cell properties and ameliorates the tumor volume

Accumulating evidence suggests that radiotherapy for cancer treatment generates ROS that mediates radiation-induced epithelial-mesenchymal transition (EMT) associated with the acquisition of CSC properties^31^. In light of the inhibitory effect of vactosertib on radiation-induced ROS generation, we examined the effect of vactosertib on pluripotent stem cell regulators, such as Yamanaka's factors (Octamer-binding transcription factor 4 (OCT4), sex determining region Y-box 2 (SOX2), Cellular myelocytomatosis (c-MYC), Kruppel-like factor 4 (KLF4)), along with mammosphere forming efficiency (MSFE) in irradiated 4T1-Luc cells. Treatment with vactosertib suppressed the mRNA expression of pluripotent stem cell regulators and MSFE induced by radiation in breast cancer cells (Fig. 5b). Furthermore, co-treatment with vactosertib suppressed the ROS generated by glucose oxidase (GOX), mRNA expression of pluripotent stem cell regulators (*Nanog* and Yamanaka's factors), and MSFE induced by GOX (Fig. 5a, c). These results suggest that vactosertib can mitigate radiation-induced CSC properties by inhibiting ROS-induced pluripotent stem cell regulators.

**Figure 6:**
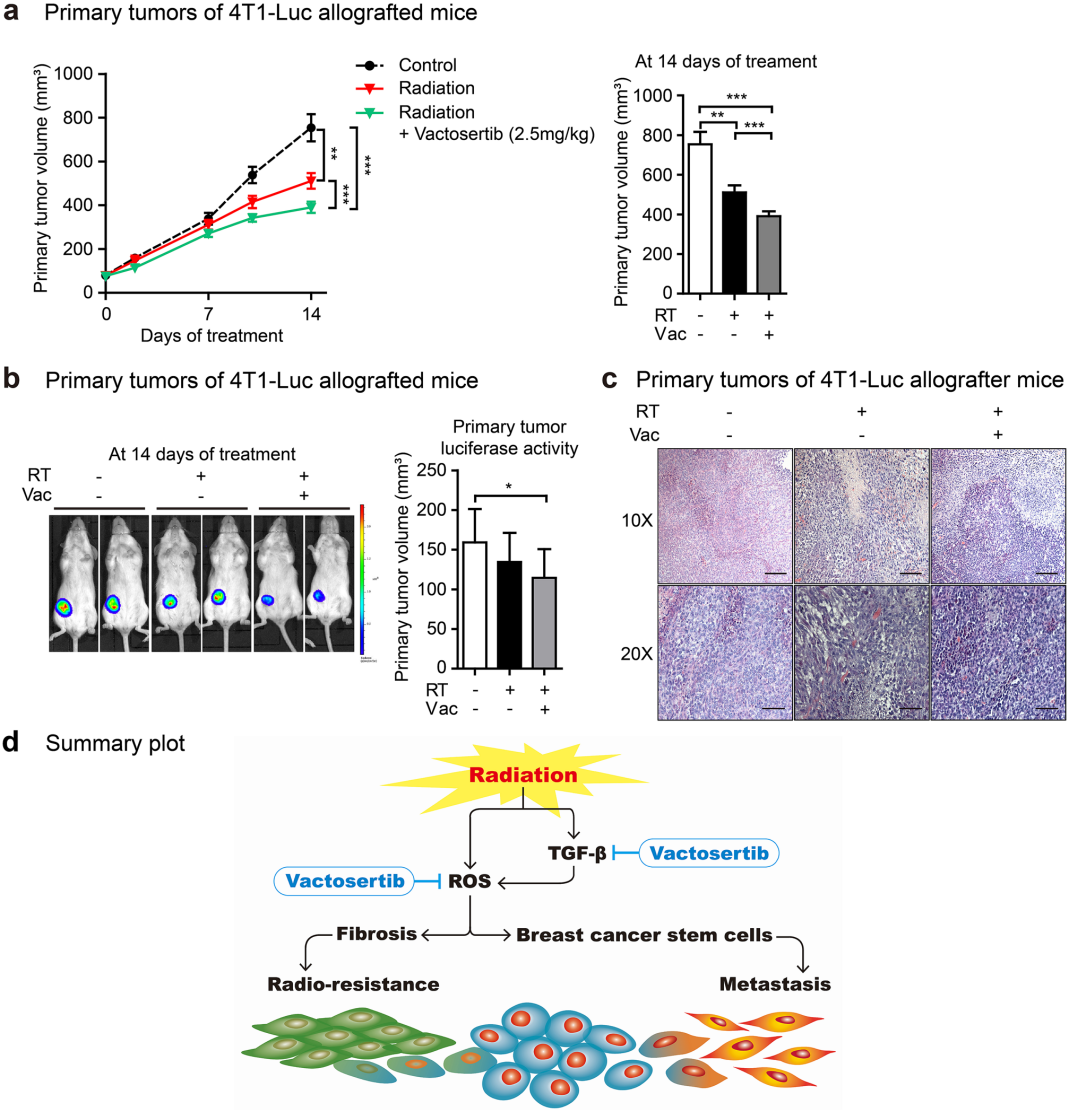
Vactosertib combination with radiotherapy ameliorates the tumor volume in a mouse model of breast cancer. (**a**) Treatment effect of vactosertib on primary tumor volume. The primary tumor volume was calculated after two weeks of treatment using the formula: volume (mm^3^) = (length (mm)) × (width (mm))^2^ × 0.5 (n = 8/group). Mice in the control group received no treatment, mice in the radiation group were irradiated with 4 Gy/day over their whole body for three days, and mice in the radiation + vactosertib group were treated vactosertib 2.5 mg/kg p.o. for two weeks and were concurrently irradiated with 4 Gy/day over their whole body for three days. (**b**) Vactosertib reduces tumor volume in irradiated BALB/c breast cancer mice. The volume of the breast tumor was measured by bioluminescence analysis. Images show the volume of representative mouse mammary tumors in each group. (**c**) H&E staining in primary tumors of mice are displayed as images showing changes in the morphology and size of tumor cells via phase-contrast microscopy (× 10 and 20, scale bar: 100 and 50 μm). (**d**) Summary plot of radiation-induced fibrosis and breast cancer stem cells. Irradiation induces transforming growth factor-β (TGF-β) and oxidative stress in breast cancer. An ALK5 inhibitor, vactosertib, suppresses TGF-β-induced fibrosis and breast cancer stem cells by inhibiting oxidative stress generated by both TGF-β-dependent and -non-dependent pathways. Vactosertib can reduce radio-resistance and metastasis in breast cancer treatment.

In addition, we investigated the effects of vactosertib on primary tumor volume of 4T1-Luc allografted mice. Although radiation monotherapy showed a slight reduction in primary tumor volume; however, co-treatment of vactosertib and radiation enhanced the anticancer effect of radiation, as evidenced by a prominent decrease of primary tumor volume (Fig. 6a). Radiotherapy combined with vactosertib reduced tumor volume compared to radiomonotherapy in the 4T1-Luc allograft mouse model (Fig. 6b). As a result of observing the cancer tissue sections, it was confirmed that the hypertrophy of cells caused by radiation was suppressed by the combination treatment with vactosertib (Fig. 6c).

## Discussion

Although radiotherapy is a major adjuvant therapeutic tool for breast cancer patients to reduce local recurrence following surgery, not all patients can benefit from radiotherapy due to various complications including fibrosis^32^. Radiotherapy-induced fibrosis worsens the quality of patient life by causing breast contracture, shoulder dysfunction, impaired cosmesis, and failure of breast reconstruction^33^. According to the clinical data, more than one-third of breast cancer patients would suffer from severe fibrosis of the reconstructed breast after radiotherapy for a long term^17,18^. In addition, radiotherapy-mediated changes in the tumor microenvironment, such as ECM remodeling; fibrosis; EMT; cancer cell stemness; damage to the vascular endothelial cells; and inflammatory response, promote radio-resistance, angiogenesis, invasion, and metastasis of cancer cells^31^. TGF-β is one of the major signaling pathways contributing to these events activated by radiation^7,31,34^. Therefore, inhibition of TGF-β signaling induced by radiotherapy has been suggested as a therapeutic target for reducing side effects of radiotherapy^19^. We reported in this study that radiation-induced TGF-β could induce fibrosis and cancer stem cell properties through ROS and that combination therapy with the TGF-β/ALK5 inhibitor vactosertib could block this series of processes (Fig. 6d). Our results showed that vactosertib could effectively block the expression of phosphorylated SMAD2/3, a major signal transducer of the radiation-induced TGF-β signaling pathway, in vitro, while at the same time repairing radiation-induced DNA damage. Although the radiation-induced SMAD2/3 phosphorylation level in vivo did not show a high expression level compared to the expression intensity in vitro, it was sufficient to confirm the phosphorylation inhibitory effect of vactosertib. Previously, it was reported that vactosertib down-regulated the expression of fibrosis markers, such as PAI-1, α-SMA, COLLAGEN, α_v_-INTEGRIN, and FIBRONECTIN, reduced ROS generation, and inhibited ECM accumulation in fibrotic disease models of liver, kidney, and lung^8^. In addition, Kim et al*.* have revealed that vactosertib repressed cholestatic liver fibrosis via inhibition of TGF-β signaling induced by hypoxia-inducible factor 1α (HIF1α) in vivo^14^. In line with these results, Soleimani et al*.* showed that vactosertib repressed the formation of fibrosis-related postsurgical adhesion through suppression of oxidative stress, profibrotic genes, and collagen deposition in vivo^35^. These findings are consistent with our results that vactosertib decreases ECM proteins such as α-SMA, COL1A1, and FIBRONECTIN, including PAI-1, which are increased in radiation-induced fibrous tissue by inhibition of TGF-β signaling, therefore, these results supported the therapeutic potential of vactosertib in the reduction of the risk of radiotherapy-induced fibrosis in cancer patients.

ROS generated by radiation plays a critical role in upregulation of TGF-β and progression of tissue fibrosis^3,4^. Then, TGF-β induces chronic oxidative stress in both direct irradiated and bystander cells and organs^4^. Previously, vactosertib attenuated fibrosis by reduction of ROS via inhibition of both Smad and non-Smad pathways in TGF-β signaling in fibrotic disease models^8^. Similarly in our results, ROS markers such as 4HNE, NOX2, and NOX4 that were increased by irradiation were attenuated by vactosertib. It has been reported that overproduction of ROS under oxidative stress conditions induces the formation of lipid peroxidation-derived products, 4-HNE, and NADPH oxidase^12^. In addition, 4-HNE increases the expression of antioxidant defense mechanism-related genes by activating Nrf2, an antioxidant master regulator, and engaging in anti-inflammatory signaling pathways^23^. We confirmed that the expression of radiation-induced antioxidant genes PRDX1, NRF2, HO-1, and NQO-1 was significantly inhibited by vactosertib, thus demonstrating the ability of vactosertib to inhibit ROS. In other study, vactosertib inhibited ROS generation in LX-2 cells (human immortalized hepatic stellate cells) treated with H_2_O_2_ or GOX or TGF-β^14^. GOX is a redox enzyme that oxidizes intracellular glucose to H_2_O_2_ to induce ROS production. We showed that in breast cancer cells, vactosertib effectively inhibited the increased ROS level by GOX, similar to the effect of Catalase, a potent ROS scavenger. ROS is a mediator of radiation-induced EMT, and EMT is also known to play an important role in the acquisition of stem cell competence in cancer cells^31^. In a previous study, we reported that radiation increased EMT markers such as Snail, Twist, and N-cadherin and that vactosertib inhibited them^19^. Based on these results, we demonstrated that the increase in pluripotent stem cell modulators induced by radiation or GOX in breast cancer cells was inhibited by vactosertib. In a previous study, vactosertib also repressed the paclitaxel-induced EMT and CSC properties of breast cancer both in vivo and in vitro^13^. Furthermore, the combination of radiotherapy and vactosertib decreased tumor volume in a mouse model of breast cancer. This indicates that vactosertib can alleviate radiation resistance and enhance the anticancer effect of radiation in the treatment of breast cancer. Various small molecules targeting TGF-β/ALK5 such as vactosertib have been examined via ongoing clinical studies with cancer patients. One of them, galunisertib (LY2157299) is being evaluated for efficacy and safety including inhibition of TGF-β signaling in advanced hepatocellular cancer patients undergoing radiotherapy through a phase I clinical study (NCT02906397). Previously, vactosertib showed more potent efficacy than galunisertib in the suppression of TGF-β-induced intracellular oxidative stress, EMT, and metastasis of breast cancer cells both in vivo and in vitro^8,12^. Clinical trials of vactosertib have been completed in the treatment of esophageal cancer, gastric cancer, biliary tract cancer, pancreatic cancer, non-small cell lung cancer, melanoma, and breast cancer, and several clinical trials of combination therapy with various agents are currently underway^36^. Therefore, based on the results of this study and the efficacy and safety profile of vactosertib, we propose that the combination treatment of vactocertib and radiation therapy will improve cancer treatment outcomes and patients' quality of life.

## Conclusion

In conclusion, this study showed that orally bioavailable TGF-β/ALK5 inhibitor, vactosertib could overcome the limitations of radiotherapy in breast cancer treatment by inhibiting oxidative stress, EMT, cancer stem cell (CSC), and fibrosis. Considering this therapeutic effect of vactosertib, co-treatment with vactosertib and radiotherapy could be clinically applicable to breast cancer patients.

## Materials and methods

### Search and review of clinical data

Clinical data for the risk of fibrosis after radiotherapy in breast cancer patients were found in the PubMed database using searches for the following keywords: breast cancer, radiotherapy, and fibrosis rate. After electronic literature search, we reviewed the collected clinical data.

### Reagents

*N*-[[4-([1,2,4]Triazolo[1,5-a]pyridin-6-yl)-5-(6-methylpyridin-2-yl)-1*H*-imidazol-2-yl]methyl]-2-fluoroaniline (Vactosertib) was synthesized by Dr. D.K. Kim (Ewha Womans University, Seoul, Korea).

### Cell culture and cell irradiation

The 4T1-Luc mouse breast cancer cells and MDA-MB-231 human breast cancer cells were obtained from the ATCC. The 4T1-Luc cells were cultured in Dulbecco's Modified Eagle's medium (DMEM) (GenDEPOT) containing Hydroxyethyl piperazine Ethane Sulfonic acid (HEPES) sodium salt (Sigma Aldrich), sodium bicarbonate (Sigma Aldrich), 5% fetal bovine serum (FBS) (GenDEPOT), and 100× penicillin–streptomycin solution (GenDEPOT). The MDA-MB-231 cells were cultured in RPMI1640 culture medium (Gibco Laboratories) containing HEPES sodium salt, sodium bicarbonate, sodium pyruvate solution (Gibco Laboratories), 5% FBS, and 100× penicillin–streptomycin solution. Cells were incubated at 37 °C in 5% CO_2_. For irradiation, cells were seeded in culture plates and were incubated with serum-reduced (0.2% FBS) medium for 16 h for starvation. Cells were pretreated with vactosertib (100 nM) for 30 min and were irradiated with 10 Gy. After 24 h of incubation, in vitro assays were conducted.

### Experimental mouse breast cancer model for co-treatment with vactosertib and radiation

Female BALB/c mice were purchased from Central Lab Animal Inc (Korea). Four to five mice were placed per cage at 21 °C room temperature with 50% humidity and a 12-h light cycle. All experimental procedures were approved by the Animal Care Committee of Ewha Womans University and complied based on the NIH Guide for the Care and Use of Laboratory Animals (Institute of Laboratory Animal Resources, National Research Council, WA). Female BALB/c mice were injected with a total of 4 × 10^4^ 4T1-Luc cells into the fourth mammary fat pad. When the tumor size was 70–100 mm^3^, mice were divided randomly into three groups (control, radiation, vactosertib + radiation group, n = 8/group). Mice in the control group received no treatment, mice in the radiation group were irradiated with 4 Gy/day over their whole body for three days, and mice in the vactosertib + radiation group were administered vactosertib 2.5 mg/kg *p.o*. for two weeks and were concurrently irradiated with 4 Gy/day over their whole body for three consecutive days. After 14 days of treatment, the breast tumor volume of surviving mice was monitored and analyzed with an in vivo imaging IVIS-200 system (Xenogen Corporation). Images were captured using the Living Image Software package (PerkinElmer / Caliper Life Sciences).

### RNA extraction and RT-PCR and qRT-PCR analyses

Total RNA from mouse tissues and cultured cells were isolated using Ambion's TRizol reagent. cDNAs were synthesized from 1 μg of total RNA using M-MLV reverse transcriptase (BIONEER) and random primers (Promega). Synthesized cDNAs were subjected to PCR amplification using SYBR Green real-time quantitative RT-PCR reagent (Applied Biosystems). The primers are listed in Supplementary Table 1. A Step-One Real-time PCR system (Applied Biosystems) was used to amplify cDNA.

### Western blot analysis

Mouse primary tumor tissues and cultured cells were homogenized in the RIPA buffer [tris–HCl (50 mM, pH 7.4), NP-40 (1%), NaCl (150 mM), sodium deoxycholate (0.5%), sodium dodecyl sulfate (0.1%), ethylene-diamine-tetraacetic acid (EDTA, 1 mM), Na3VO4 (1 mM), NaF (50 mM), phenylmethylsulfonyl fluoride (1 mM), protease inhibitor cocktail (Roche)] for 30 min on ice. The lysate was centrifugated at 13,000 rpm and 4 °C for 10 min. The protein content of the lysates was determined using a Bicinchoninic acid (BCA) protein assay kit (Thermofisher Scientific). Protein samples (5–10 μg) of tissues and cells were separated by sodium dodecyl sulfate–polyacrylamide gel (6–12%) electrophoresis. Transfer to polyvinylidene fluoride (Millipore) or nitrocellulose (Whatman) membranes and block with 5% bovine serum albumin (BSA) (GenDEPOT) or 5% dry skim milk (BD Biosciences). Primary antibodies (Supplementary Table 2) were incubated for 1 h at room temperature and then washed in 1× Tris-buffered saline containing 0.1% Tween 20 detergent. Membranes were incubated with horseradish peroxidase-conjugated (HRP) secondary antibody for 30 min and then washed with 1X TBS. Proteins were detected using ATTO (Enhanced chemiluminescence kit), and band intensity was analyzed by LAS-3000 densitometer (Fujifilm).

### Fluorescence immunohistochemistry (IHC) assay

For detecting PAI-1(Plasminogen activator inhibitor-1), 4-hydroxy nonenal (4-HNE), NOX4, α-smooth muscle actin (α-SMA), Collagen Type I Alpha 1 Chain (COL1A1), and p-SMAD2/3 by fluorescence immunohistochemistry assay, formalin-fixed and paraffin-embedded sections of mouse tissues were dewaxed in an OTTIX bath (Diapath) and were blocked with solution containing 5% BSA (GenDEPOT) and 0.1% Triton 100× (Merky). Slides were incubated with a primary antibody mixture of anti-PAI-1 rabbit IgG (Santa Cruz Biotechnology), anti-4-HNE rabbit IgG (Abcam), and anti-NOX4 rabbit IgG (Santa Cruz Biotechnology) or with a mixture of anti-α-SMA mouse IgG (Sigma Aldrich), anti-COL1A1 mouse IgG (Santa Cruz Biotechnology), and anti-p-SMAD2/3 mouse IgG (Santa Cruz Biotechnology). Then, a mixture of Alexa 488-conjugated anti-rabbit IgG (Cell Signaling) F(ab’) fragments was used for visualization of PAI-1, 4-HNE, and NOX4 proteins. A mixture of Alexa 488-conjugated anti-mouse IgG (Cell Signaling) F(ab’) fragments was used for visualization of α-SMA, COL1A1, and p-SMAD2/3 proteins. Mouse tissues were counterstained with 4′6-diamidino-2-phenylindole (DAPI). Fluorescence was visualized using an Axio inverted microscope (Carl Zeiss).

### Hematoxylin and eosin (H&E) staining

Formalin-fixed and paraffin-embedded sections of primary tumors from each group were dewaxed in an OTTIX bath (Diapath) and followed by hematoxylin (Sigma Aldrich) and eosin (Diapath). Tumor section images were shown by phase-contrast microscopy (Carl Zeiss).

### Immunofluorescence (IF) assay

For in vitro assay, cells were seeded on cover glass in 6 cm plates and were incubated for 24 h for attachment. The cells were incubated with serum-reduced (0.2% FBS) medium for 16 h for starvation and irradiated with 10 Gy with or without 30-min pretreatment with 100 nM vactosertib. After 24 h, cells were fixed with 4% formaldehyde solution (pH 7.4) and were blocked in 5% BSA with normal serum in 0.1% Triton 100X. Then, cells were incubated with primary and secondary antibodies. Anti-4-HNE rabbit IgG (Abcam) and Alexa 488-conjugated anti-rabbit IgG (Cell Signaling) F(ab’) fragments were used as primary and secondary antibody, respectively. Cells were counterstained with DAPI. Fluorescence was visualized using an Axio inverted microscope (Carl Zeiss).

### ROS assay

Intracellular accumulation of ROS was measured using the membrane permeable fluorescent dye 2',7' dichlorodihydrofluorescein diacetate (H_2_DCFDA) (Invitrogen). The H_2_DCFDA was converted to a membrane impermeable highly fluorescent compound, dichlorofluorescin diacetate (DCF), by intracellular esterase and ROS. Cells were seeded on 6 cm cell culture plates and were incubated for 24 h with complete medium. The cells were incubated with serum-reduced (0.2% FBS) medium for 16 h for starvation. For a measure of ROS level, cells were treated with vactosertib (100 nM), glucose oxidase (GOX, 2.5 mU/ml), catalase (CAT, 500 U/ml), vactosertib (100 nM) + GOX (2.5 mU/ml), or vactosertib (100 nM) + CAlT (500 U/ml). Then, cells were treated with H_2_DCFDA (10 mM), and the level of ROS was measured using a Microplate Multi-Reader (Infinite F200 PRO) at excitation: 495 nm/emission: 525 nm.

### Mammosphere-forming assay

For a mammosphere forming assay by radiation-induced ROS, cells were seeded in 10 cm plates and were incubated for 24 h for attachment. Cells were incubated with serum-reduced (0.2% FBS) medium for 16 h for starvation and irradiated with 10 Gy with or without 30-min pretreatment with 100 nM vactosertib. After 24 h of incubation, we harvested the adherent cells with trypsin–EDTA and reseeded the cells in ultra-low attachment dishes. Cells were incubated in DMEM/F-12 (1:1) (GenDEPOT) supplemented with B-27 Supplement (50X) (Gibco Laboratories), 10 ng/ml basic fibroblast growth factor (bFGF) (Invitrogen), and 10 ng/ml Epidermal Growth Factor (EGF) (Sigma Aldrich). To measure mammosphere-forming efficiency (MSFE), the number of spheres (> 50 μm) was counted after one week. MSFE indicates the number of spheres divided by the original number of cells seeded and is presented as %.

For a mammosphere forming assay by GOX-generated ROS, cells were seeded in ultra-low attachment dishes and incubated in DMEM/F-12 (1:1) (GenDEPOT) supplemented with B-27 Supplement (50X) (Gibco Laboratories), 10 ng/ml bFGF (Invitrogen), and 10 ng/ml EGF (Sigma Aldrich). Cells were treated with 2.5 mU/ml GOX with or without co-treatment with 100 nM vactosertib. To measure MSFE, the number of spheres (> 100 μm) was counted after one week.

### Statistical analysis

The data for results are expressed as mean ± standard error of the mean (SEM). Data represent the means of three independent experiments performed in triplicate. Statistical values were determined by one-way analysis of variance (ANOVA) with the Bonferroni post-hoc test. Each asterisks are used to indicate statistical significance (*, **, and *** indicate *p* < 0.05, *p* < 0.01, and *p* < 0.005, respectively).

### Ethics approval and consent to participate

All animal experimental procedures were approved by the Animal Care Committee of Ewha Womans University. All methods were carried out in accordance with relevant guidelines and regulations. All methods are reported in accordance with ARRIVE guidelines (https://arriveguidelines.org).

## Supplementary Information

Supplementary Information 1.

Supplementary Information 2.

## Data Availability

All data generated or analyzed during this study are included in this published article (and its Supplementary Information files). The data that support the findings of this study are available from Johannes Claßen MD et al., but restrictions apply to the availability of these data, which were used under license for the current study, and so are not publicly available. Data are available from the authors upon reasonable request and with permission.
