# Supplementary material for: Radiotherapy-induced oxidative stress and fibrosis in breast cancer are suppressed by vactosertib, a novel, orally bioavailable TGF-β/ALK5 inhibitor
Source: Sci Rep. 2022 Sep 27;12:16104. doi: 10.1038/s41598-022-20050-9 (PMC9515166; doi:10.1038/s41598-022-20050-9)
Supplement: Supplementary file 1 — Supplementary Information 1. [file 41598_2022_20050_MOESM1_ESM.docx]

**Radiotherapy-induced oxidative stress and fibrosis in breast cancer are suppressed by vactosertib, a novel, orally bioavailable TGF-β/ALK5 inhibitor**

Jiyoung Park ^1,2 #^, Jiwon Choi ^1,2 #^, Ilyoung Cho ^1 #^, Yhun Yhong Sheen ^1*^

^1^College of Pharmacy, Ewha Womans University, 52, Ewhayeodae-gil, Seodaemun-gu, Seoul, 03760, Republic of Korea

Current address: ^2^National Center for Efficacy Evaluation for Respiratory Disease Products, Korea Institute of Toxicology, 30 Baehak1-gil, Jeongeup, Jeollabuk-do, 56212, Republic of Korea.

**Supplementary Figure**


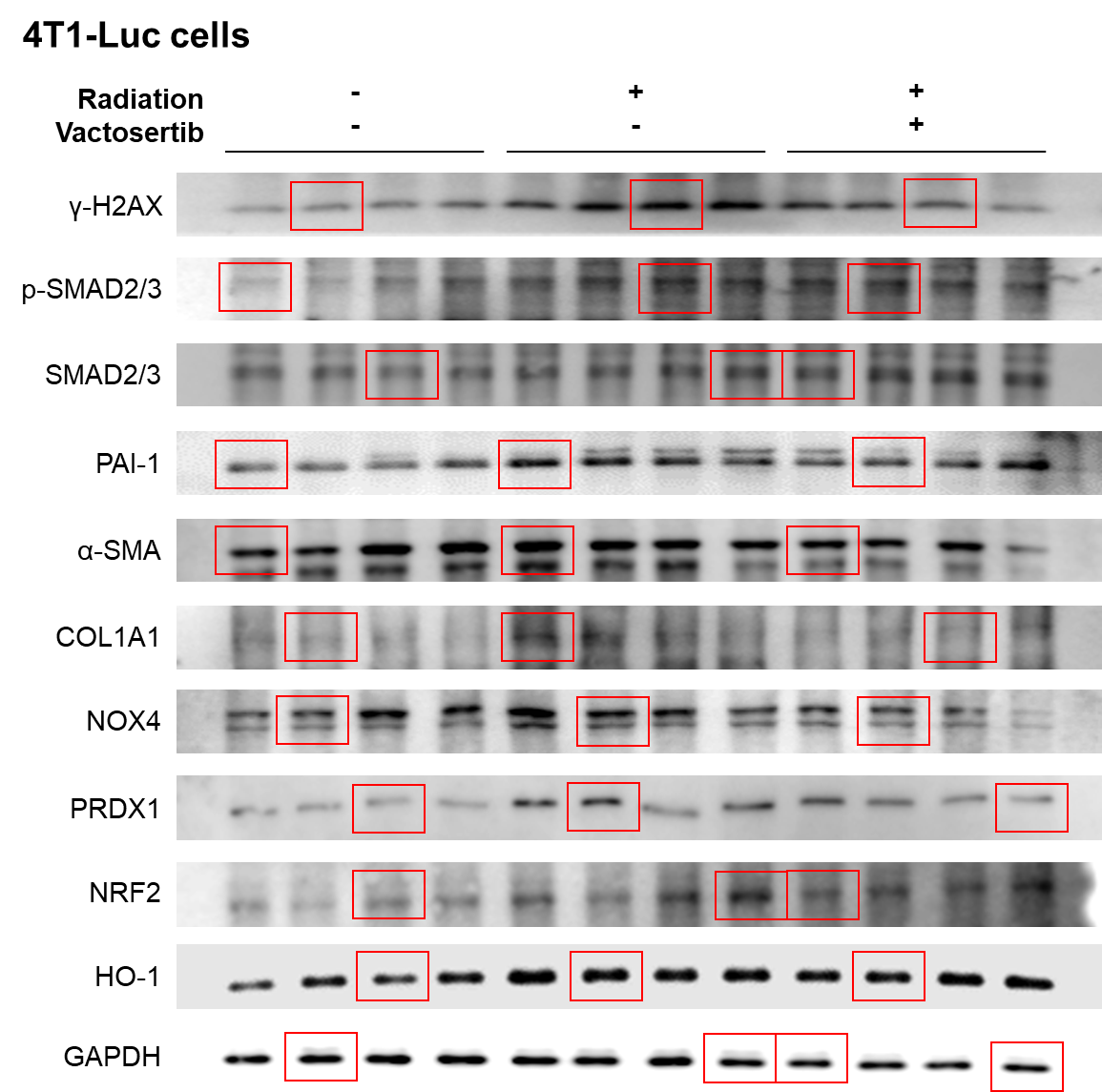


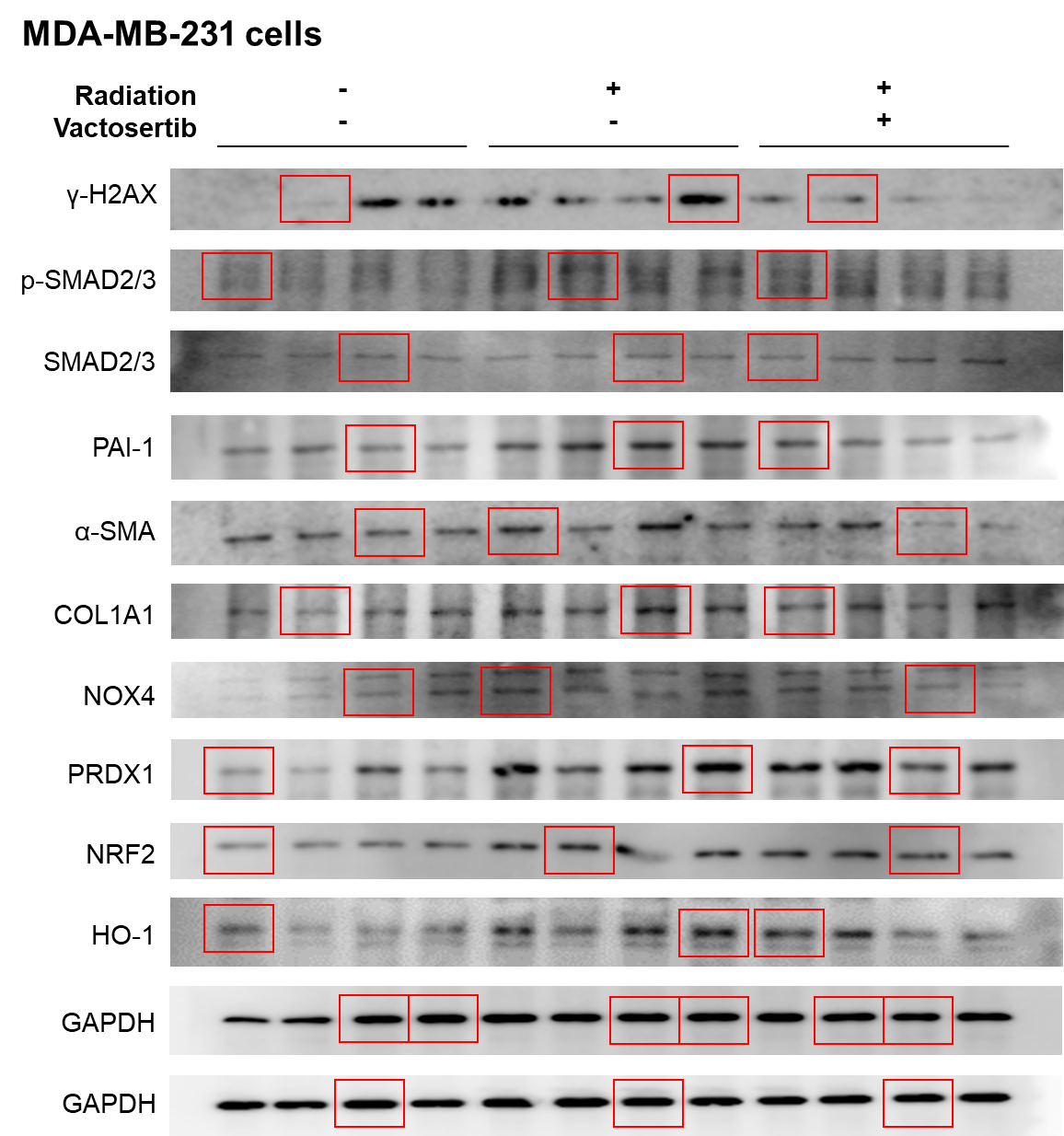


**Supplementary figure 1.** **Original western blot for protein level in the 4T1-Luc cells (upper panel) and MDA-MB-231 cells (down panel).** Full-length western blot for 4T1-Luc cells and MDA-MB-231 cells corresponding to Figures 1 to 4. Samples from each group were loaded in quadruplicate. All bands were respectively quantified with GAPDH. Representative bands in the whole blot are indicated by red boxes. The whole membrane image is shown on the back.

**
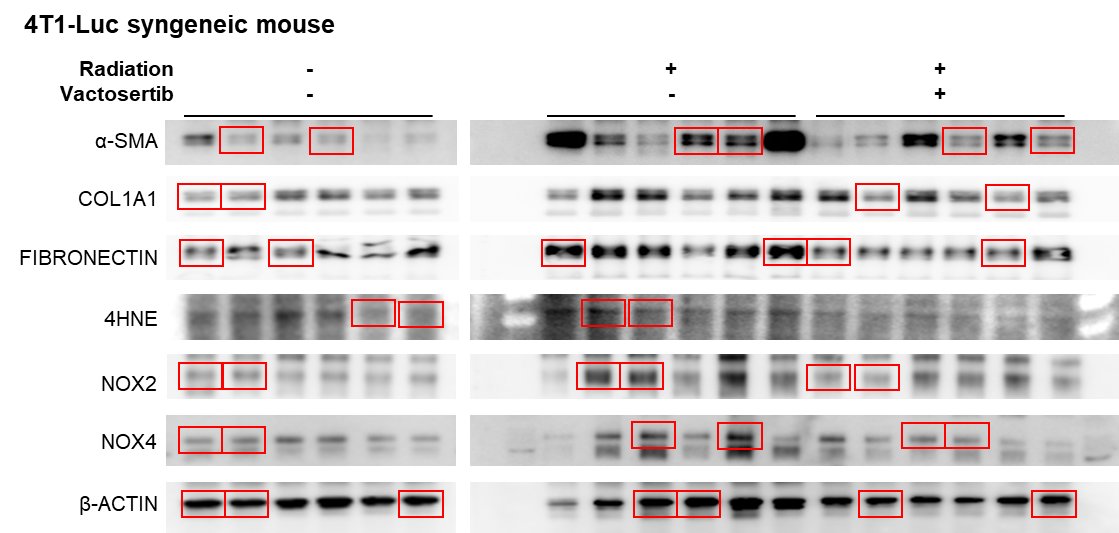
**

**Supplementary figure 2.** **Original western blot for primary tumor of 4T1-Luc syngeneic mouse.** Full-length western blot for mouse primary tumor tissues corresponding to Figures 1 to 4. Primary tumor tissue samples from mice were loaded 6 in each group. All bands were respectively quantified with β-ACTIN. Representative bands in the whole blot are indicated by red boxes. The whole membrane image is shown on the back.

**
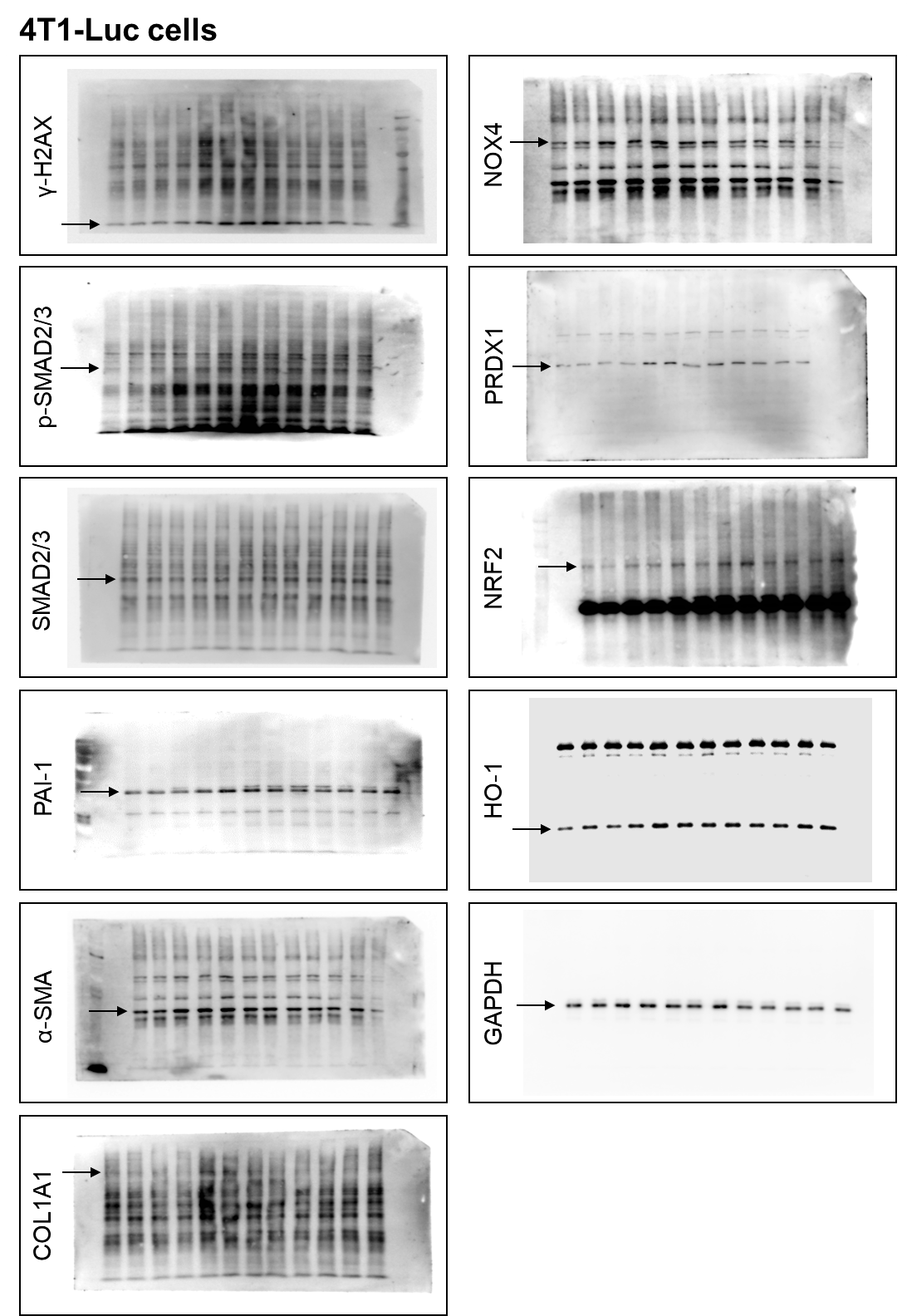

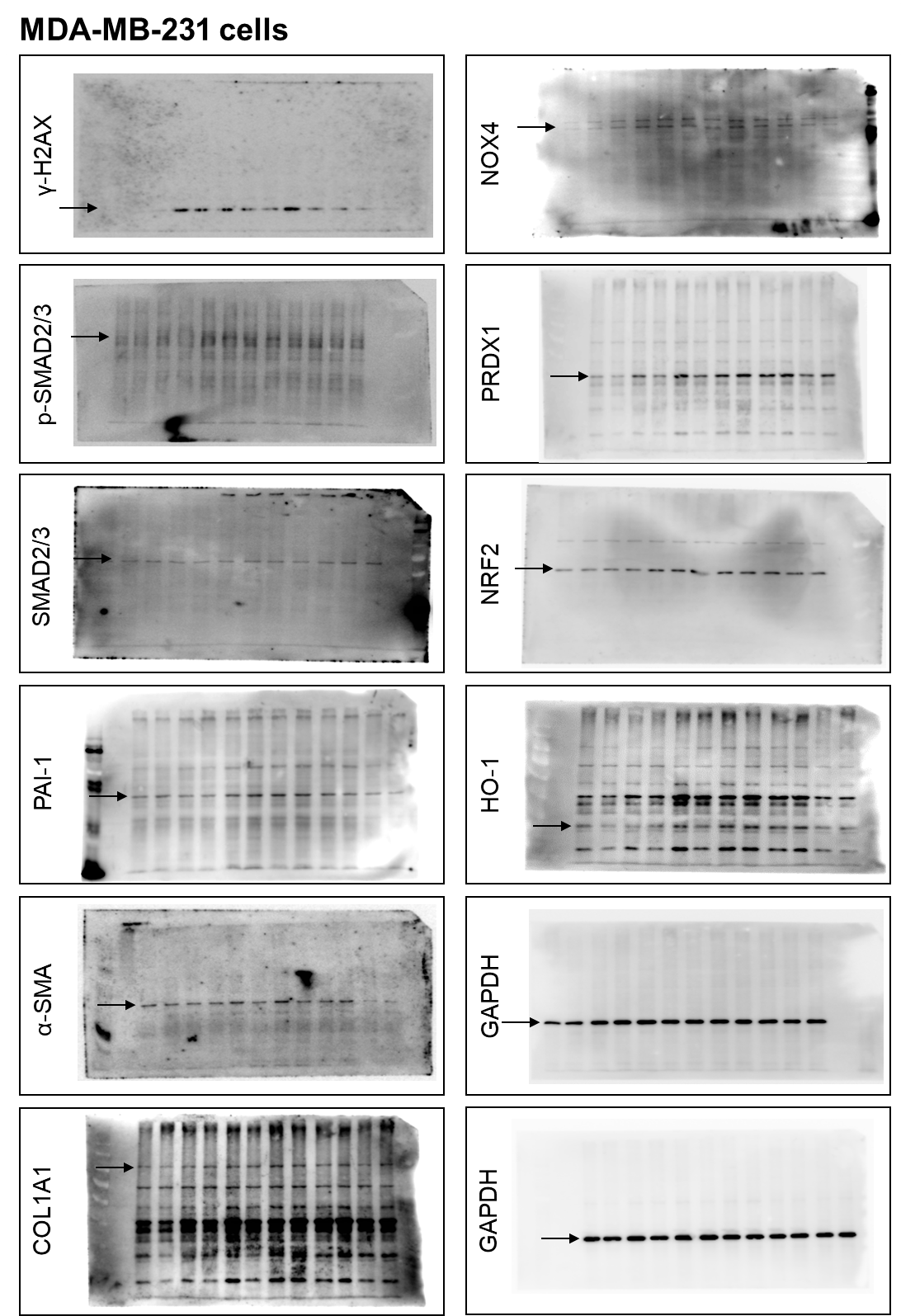

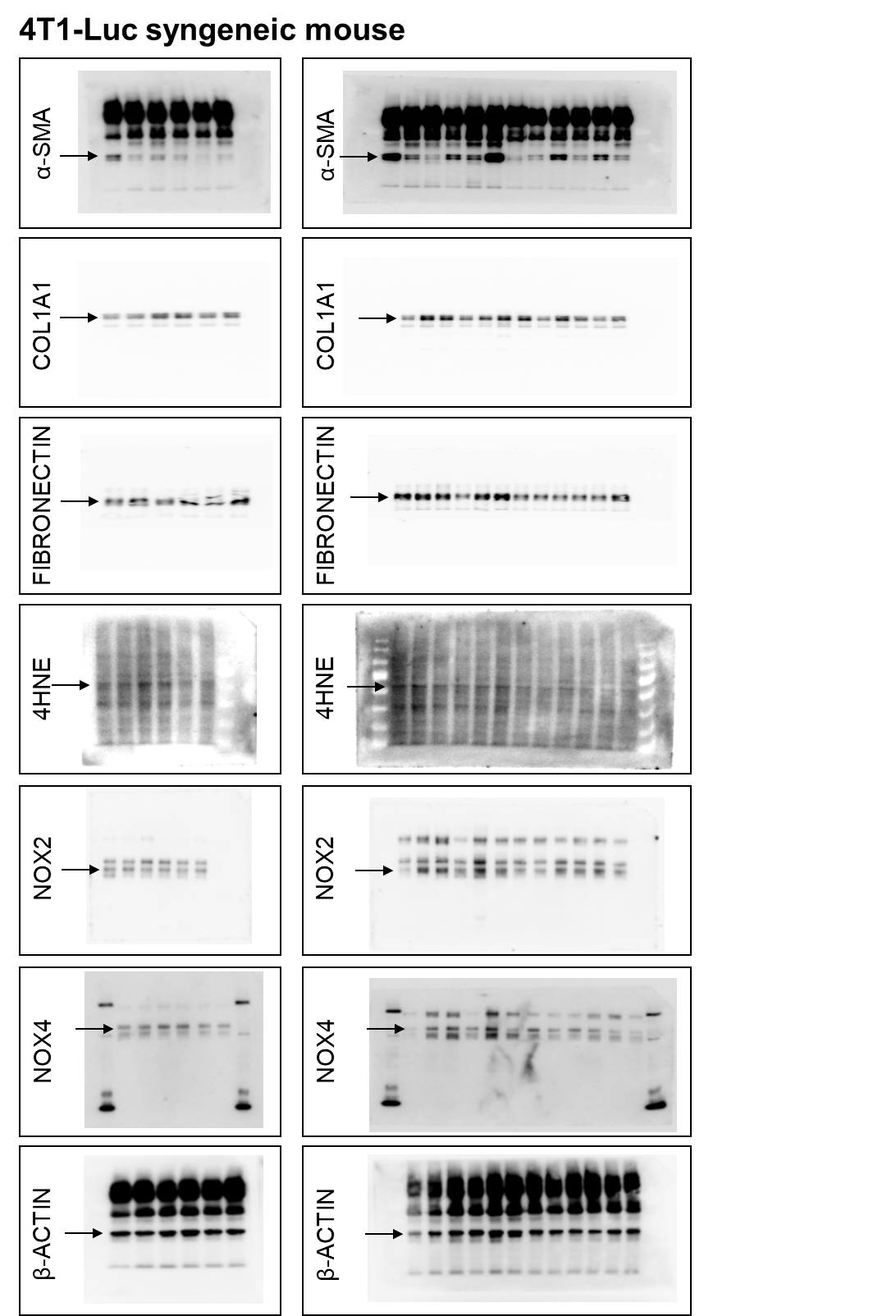
**
