# Supplementary material for: Radiotherapy-induced oxidative stress and fibrosis in breast cancer are suppressed by vactosertib, a novel, orally bioavailable TGF-β/ALK5 inhibitor
Source: Sci Rep. 2022 Sep 27;12:16104. doi: 10.1038/s41598-022-20050-9 (PMC9515166; doi:10.1038/s41598-022-20050-9)
Supplement: Supplementary file 2 — Supplementary Information 2. [file 41598_2022_20050_MOESM2_ESM.docx]

**Radiotherapy-induced oxidative stress and fibrosis in breast cancer are suppressed by vactosertib, a novel, orally bioavailable TGF-β/ALK5 inhibitor**

Jiyoung Park ^1,2 #^, Jiwon Choi ^1,2 #^, Ilyoung Cho ^1 #^, Yhun Yhong Sheen ^1*^

^1^College of Pharmacy, Ewha Womans University, 52, Ewhayeodae-gil, Seodaemun-gu, Seoul, 03760, Republic of Korea

Current address: ^2^National Center for Efficacy Evaluation for Respiratory Disease Products, Korea Institute of Toxicology, 30 Baehak1-gil, Jeongeup, Jeollabuk-do, 56212, Republic of Korea.

**Supplementary Table**

**Supplementary Table 1. Primers used for qRT-PCR**

| Gene | Forward (5’ – 3’) | Reverse (5’ – 3’) |
| --- | --- | --- |
| *Pai-1* | CCTCATCCTGGGCCTGGTTCTGGTCT | GGTTTTCCCCGCTGTGGTCATCTGC |
| *α-sma* | GACGCTGAAGTATCCGATAGAACACG | CACCATCTCCAGAGTCCAGCACAAT |
| *Col1a1* | ACCTGTGTGTTCCCTACTCA | GACTGTTGCCTTCGCCTCTG |
| *Nox4* | TCTGTTGTGGACCCAATTCA | AGCTGATTGATTCCGCTGAG |
| *Nrf2* | GCCCACATTCCCAAACAAGAT | CCAGAGAGCTATTGAGGGACTG |
| *Ho-1* | AAGCCGAGAATGCTGAGTTCA | GCCGTGTAGATATGGTACAAGGA |
| *Nqo-1* | AGGATGGGAGGTACTCGAATC | AGGCGTCCTTCCTTATATGCTA |
| *Nanog* | TGGGATTTACAGGCGTGAGC | AAGCAAAGCCTCCCAATCCC |
| *Oct4* | GGGCTCTCCCATGCATTCAA | CACCTTCCCTCCAACCAGTT |
| *Sox2* | TCGGCAGACTGATTCAAATAATACAG | CCATGCAGGTTGACACCGTTG |
| *Klf4* | ACGATCGTGGCCCCGGAAAA | CAACAACCGAAAATGCACCAGCCCCA |
| *Myc (c-Myc)* | GCGTCCTGGGAAGGGAGATCCGGAGC | TTGAGGGGCATCGTCGCGGGAGGCTG |
| *Ppia* | TGCACAGACGGTCACTCAAA | TGCCATCGCCAAGGAGTAG |
| *Hprt* | CTTTGCTTTCCTTGGTCAGG | GGTCCTTTTCACCAGCAAGC |

**Supplementary Table 2. Antibodies for Western blot analysis**

**2.1. Primary Antibodies**

| Antibody | Company | Product number |
| --- | --- | --- |
| p-SMAD2/3 | Santa Cruz Biotechnology, Santa Cruz, CA, USA | sc-11769 |
| SMAD2/3 | BD Biosciences, San Diego, CA, USA | 610842 |
| γ-H2AX | Millipore, Billerica, MA, USA | 2652964 |
| PAI-1 | Santa Cruz Biotechnology, Santa Cruz, CA, USA | sc-8979 |
| α-SMA | Sigma Aldrich, St. Louis, MO, USA | A2547 |
| COL1A1 | Santa Cruz Biotechnology, Santa Cruz, CA, USA | sc-59772 |
| FIBRONECTIN | BD Biosciences, San Diego, CA, USA | 610077 |
| 4HNE | Abcam, Cambridge, MA, USA | ab46545 |
| NOX2 | Santa Cruz Biotechnology, Santa Cruz, CA, USA | sc-5827 |
| NOX4 | Santa Cruz Biotechnology, Santa Cruz, CA, USA | sc-30141 |
| PRDX1 | Supplied by Dr. H.A. Woo (Ewha Womans University, Seoul, Korea) | |
| NRF2 | Santa Cruz Biotechnology, Santa Cruz, CA, USA | sc-365949 |
| HO-1 | Santa Cruz Biotechnology, Santa Cruz, CA, USA | sc-136960 |
| GAPDH | Sigma Aldrich, St. Louis, MO, USA | MAB374 |
| β-ACTIN | Sigma Aldrich, St. Louis, MO, USA | A5316 |

**2.2 Secondary Antibodies**

| Antibody | Company | Product number |
| --- | --- | --- |
| Anti-rabbit HRP | Gendepot, Barker, TX, USA | SA002-500 |
| Anti-mouse HRP | Gendepot, Barker, TX, USA | SA001-500 |
| Anti-goat HRP | Santa Cruz Biotechnology, Santa Cruz, CA, USA | sc-2768 |
